# Tris(1,10-phenanthroline-κ^2^ *N*,*N*′)nickel(II) bis­(2,4,5-tricarb­oxy­benzo­ate) monohydrate

**DOI:** 10.1107/S1600536811048914

**Published:** 2011-11-23

**Authors:** Kai-Long Zhong, Chao Ni, Ming-Yi Qian

**Affiliations:** a Department of Applied Chemistry, Nanjing College of Chemical Technology, Nanjing 210048, People’s Republic of China

## Abstract

In the title compound, [Ni(C_12_H_8_N_2_)_3_](C_10_H_5_O_8_)_2_·H_2_O, the Ni^II^ cation is coordinated by six N atoms of the three bidentate chelating 1,10-phenanthroline ligands in a slightly distorted octa­hedral coordination geometry. The Ni—N bond lengths range from 2.074 (2) to 2.094 (2) Å. The dihedral angles between the three chelating NCCN groups to each other are 85.71 (3), 73.75 (2) and 85.71 (3)°, respectively. The Ni cation, the phenyl ring of the 1,10-phenanthroline ligand and the lattice water molecule are located on special positions (site symmetry 2). In the crystal, the uncoordinated 2,4,5-tricarb­oxy­benzeno­ate anions join with each other through O—H⋯O hydrogen bonds, forming a two-dimensional hydrogen-bonded layer structure along the *bc* plane. The layers are further linked *via* additional O—H⋯O inter­actions between water and carboxyl groups, resulting in a three-dimensional supra­molecular network.

## Related literature

For structures of complexes with six-coordinate nickel atoms and background references, see: Li *et al.* (2003 ▶); Fu *et al.* (2004 ▶); Fabelo *et al.* (2008 ▶); Zhong *et al.* (2009 ▶); Ni *et al.* (2010 ▶). For background to phenanthroline complexes, see: Wang & Zhong (2011 ▶); Zhu *et al.* (2006 ▶); Cui *et al.* (2010 ▶); Zhong (2011*a* ▶,*b* ▶,*c* ▶). 
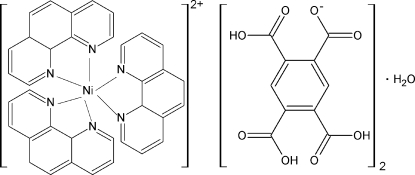


## Experimental

### 

#### Crystal data

[Ni(C_12_H_8_N_2_)_3_](C_10_H_5_O_8_)_2_·H_2_O
*M* *_r_* = 1123.62
Monoclinic, 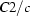

*a* = 24.2009 (11) Å
*b* = 14.1546 (5) Å
*c* = 15.8347 (7) Å
β = 116.271 (5)°
*V* = 4864.0 (4) Å^3^
*Z* = 4
Mo *K*α radiation
μ = 0.49 mm^−1^
*T* = 295 K
0.40 × 0.40 × 0.30 mm

#### Data collection

Oxford Diffraction Xcalibur Sapphire3 Gemini ultra diffractometer
Absorption correction: multi-scan (*ABSPACK*; Oxford Diffraction, 2009 ▶) *T*_min_ = 0.829, *T*_max_ = 0.868
12270 measured reflections
4977 independent reflections
3050 reflections with *I* > 2σ(*I*)
*R*_int_ = 0.037

#### Refinement

*R*[*F* ^2^ > 2σ(*F* ^2^)] = 0.041
*wR*(*F* ^2^) = 0.054
*S* = 1.02
4977 reflections
369 parameters
3 restraints
H atoms treated by a mixture of independent and constrained refinement
Δρ_max_ = 0.52 e Å^−3^
Δρ_min_ = −0.42 e Å^−3^

### 

Data collection: *CrysAlis PRO* (Oxford Diffraction, 2009 ▶); cell refinement: *CrysAlis PRO*; data reduction: *CrysAlis PRO*; program(s) used to solve structure: *SHELXS97* (Sheldrick, 2008 ▶); program(s) used to refine structure: *SHELXL97* (Sheldrick, 2008 ▶); molecular graphics: *XP* in *SHELXTL* (Sheldrick, 2008 ▶); software used to prepare material for publication: *SHELXTL*.

## Supplementary Material

Crystal structure: contains datablock(s) global, I. DOI: 10.1107/S1600536811048914/bq2318sup1.cif

Structure factors: contains datablock(s) I. DOI: 10.1107/S1600536811048914/bq2318Isup2.hkl

Additional supplementary materials:  crystallographic information; 3D view; checkCIF report

## Figures and Tables

**Table 1:** Hydrogen-bond geometry (Å, °)

*D*—H⋯*A*	*D*—H	H⋯*A*	*D*⋯*A*	*D*—H⋯*A*
O4—H4⋯O5^i^	0.82	1.87	2.690 (2)	177
O1—H5⋯O6^ii^	0.82	1.81	2.624 (2)	173
O7—H7⋯O6	0.82	1.63	2.4450 (19)	178
O1*W*—H1*WA*⋯O2^iii^	0.80 (2)	2.10 (3)	2.866 (3)	158 (4)

Symmetry codes: (i) 
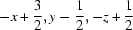
; (ii) 
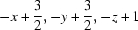
; (iii) 
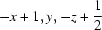
.

**Fig. 1.**
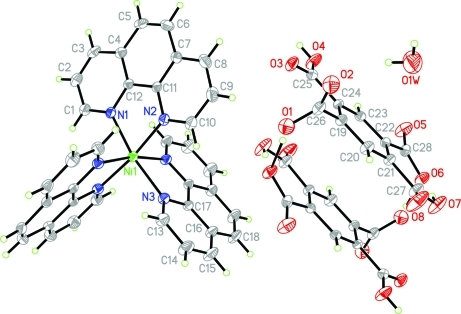
The unit of (I), showing the atom-numbering scheme and with displacement ellipsoids drawn at the 35% probability level. Unlabeled atoms of [Ni(C12H8N2)3]2+ and C10H5O8- anion are related to the labeled atoms by the symmetry operator -x + 2, y, -z + 3/2 and -x + 3/2, -y + 3/2, -z + 1, respectively.

**Fig. 2.**
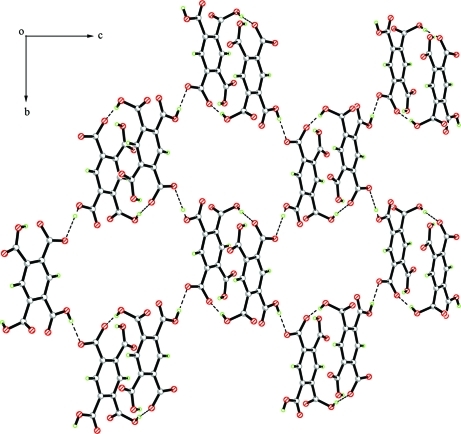
Hydrogen-bonding interaction of (I), viewed along the a axis. Dashed lines indicate hydrogen bonds. All cations [Ni(C12H8N2)3]2+ and water molecules have been omitted for clarity.

**Fig. 3.**
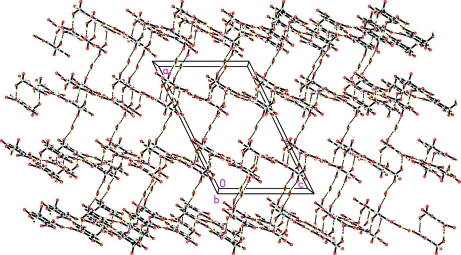
The packing, viewed down the b axis. Dashed lines indicate hydrogen bonds. All cations [Ni(C12H8N2)3]2+ have been omitted for clarity.

## supplementary crystallographic information

### Comment

1,10-Phenanthroline (Phen) and 1,2,4,5-Benzenetetracarboxylate have also been widely employed as polydentate ligands in coordination reactions and in the construction of supermolecular networks (Li *et al.*, 2003; Fu *et al.*, 2004; Fabelo *et al.*, 2008). Recently we have synthesized and reported many metal-Phen complexes such as cadmium complexe (Zhong, 2011*a*), cobalt complexes (Wang & Zhong, 2011), copper complexes (Zhong 2011*b*,*c*), nickel complexes (Zhong *et al.*, 2009; Ni *et al.*, 2010), manganese complex (Zhu *et al.*, 2006), and zinc complex (Cui *et al.*, 2010). The title compound [Ni(C_12_H_8_N_2_)_3_](C_10_H_5_O_8_)_2_.H_2_O, (I) was obtained unintentionally during an attempt to synthesize a mixed-ligand complex of Ni^II^ with Phen and 1,2,4,5-benzenetetracarboxylate ligand *via* a hydrothermal (solvothermal) reaction. The crystal structure of (I), has not hitherto been reported.

X-ray diffraction indicated that the title compound, (I), has the Ni^2+^ metal ion in a slightly distorted octahedral coordination geometry. The Ni^II^ atom is bonded by six N atoms of the three bidentate chelating 1,10-phenanthroline ligands. In the cation of [Ni(phen)_3_]^2+^, the Ni—N bond distances range from 2.074 (2) Å to 2.094 (2) Å and the N—Ni—N bite angles [80.02 (7)–79.49 (9)°] (see Table 1), which are similar to the reported literature values (Zhong *et al.*, 2009; Ni *et al.*, 2010). The dihedral angles between the neighbor two chelating NCCN groups is 85.71 (3)°, 73.75 (2)° and 85.71 (3)°, respectively. A twofold rotation axis (symmetry code: -*x* + 2, *y*, -*z* + 3/2) passes through the Ni atom and the phenyl ring of 1,10-phenanthroline. In the crystal structure, the uncoordinated trihydrogen-1,2,4,5-benzenetetracarboxylate anions (C_10_H_5_O_8_^-^) connected to each other by intermolecular  O—H···O H-bonds through carboxylic acid to form a two-dimensional hydrogen-bonded layer structure along *bc* plane. The adjacent layers are further linked *via* additional water O–H···O carboxyl hydrogen interactions, forming a three-dimensional supramolecular network structure.

### Experimental

0.1 mmol NiSO_4_^.^7H_2_O, 0.1 mmol phen, 0.1 mmol 1,2,4,5-Benzenetetracarboxylic acid and 3.0 ml water were mixed and placed in a thick Pyrex tube, which was sealed and heated to 423 K for 96 h, whereupon orange block-shaped crystals of (I) were obtained.

### Refinement

The H atoms of Phen and trihydrogen-1,2,4,5-benzenetetracarboxylate were positioned geometrically and allowed to ride on their parent atoms, with C—H = 0.93 Å; O—H = 0.82 Å and *U*_iso_(H) = 1.2*U*_eq_(C); *U*_iso_(H) = 1.5*U*_eq_(O). The H atoms of water were located in difference map and then allowed to ride on their parent atoms, with O—H = 0.81 Å and 1.5*U*_eq_(O).

### Crystal data

**Table:**

[Ni(C_12_H_8_N_2_)_3_](C_10_H_5_O_8_)_2_·H_2_O	*F*(000) = 2312
*M**_r_* = 1123.62	*D*_x_ = 1.534 Mg m^−^^3^
Monoclinic, *C*2/*c*	Mo *K*α radiation, λ = 0.71073 Å
Hall symbol: -C 2yc	Cell parameters from 4069 reflections
*a* = 24.2009 (11) Å	θ = 2.8–29.2°
*b* = 14.1546 (5) Å	µ = 0.49 mm^−^^1^
*c* = 15.8347 (7) Å	*T* = 295 K
β = 116.271 (5)°	Block, orange
*V* = 4864.0 (4) Å^3^	0.40 × 0.40 × 0.30 mm
*Z* = 4	

### Data collection

**Table:**

Oxford Diffraction Xcalibur Sapphire3 Gemini ultra diffractometer	4977 independent reflections
Radiation source: Enhance (Mo) X-ray Source	3050 reflections with *I* > 2σ(*I*)
graphite	*R*_int_ = 0.037
Detector resolution: 15.9149 pixels mm^-1^	θ_max_ = 26.4°, θ_min_ = 2.9°
ω scans	*h* = −30→29
Absorption correction: multi-scan (ABSPACK; Oxford Diffraction, 2009)	*k* = −14→17
*T*_min_ = 0.829, *T*_max_ = 0.868	*l* = −14→19
12270 measured reflections	

### Refinement

**Table:**

Refinement on *F*^2^	Primary atom site location: structure-invariant direct methods
Least-squares matrix: full	Secondary atom site location: difference Fourier map
*R*[*F*^2^ > 2σ(*F*^2^)] = 0.041	Hydrogen site location: inferred from neighbouring sites
*wR*(*F*^2^) = 0.054	H atoms treated by a mixture of independent and constrained refinement
*S* = 1.02	*w* = 1/[σ^2^(*F*_o_^2^) + (0.0092*P*)^2^] where *P* = (*F*_o_^2^ + 2*F*_c_^2^)/3
4977 reflections	(Δ/σ)_max_ < 0.001
369 parameters	Δρ_max_ = 0.52 e Å^−^^3^
3 restraints	Δρ_min_ = −0.42 e Å^−^^3^

### Special details

**Table:**

Geometry. All e.s.d.'s (except the e.s.d. in the dihedral angle between two l.s. planes) are estimated using the full covariance matrix. The cell e.s.d.'s are taken into account individually in the estimation of e.s.d.'s in distances, angles and torsion angles; correlations between e.s.d.'s in cell parameters are only used when they are defined by crystal symmetry. An approximate (isotropic) treatment of cell e.s.d.'s is used for estimating e.s.d.'s involving l.s. planes.
Refinement. Refinement of *F*^2^ against ALL reflections. The weighted *R*-factor *wR* and goodness of fit *S* are based on *F*^2^, conventional *R*-factors *R* are based on *F*, with *F* set to zero for negative *F*^2^. The threshold expression of *F*^2^ > σ(*F*^2^) is used only for calculating *R*-factors(gt) *etc*. and is not relevant to the choice of reflections for refinement. *R*-factors based on *F*^2^ are statistically about twice as large as those based on *F*, and *R*- factors based on ALL data will be even larger.

### Fractional atomic coordinates and isotropic or equivalent isotropic displacement parameters (Å^2^)

**Table:**

	*x*	*y*	*z*	*U*_iso_*/*U*_eq_	
Ni1	1.0000	0.31042 (3)	0.7500	0.03040 (12)	
N1	1.02407 (7)	0.21222 (12)	0.67422 (11)	0.0312 (5)	
N2	0.91737 (7)	0.30145 (13)	0.62879 (11)	0.0326 (4)	
N3	0.97131 (7)	0.42419 (13)	0.80545 (10)	0.0318 (5)	
O1	0.68321 (7)	0.46426 (12)	0.52192 (11)	0.0511 (4)	
H5	0.7152	0.4948	0.5473	0.077*	
O1W	0.5000	0.5789 (3)	0.2500	0.1201 (14)	
H1WA	0.4734 (13)	0.541 (2)	0.223 (3)	0.169 (19)*	
O2	0.59675 (7)	0.46988 (12)	0.39080 (11)	0.0560 (5)	
O3	0.70784 (6)	0.44147 (11)	0.35063 (10)	0.0452 (4)	
O4	0.74751 (8)	0.53534 (11)	0.27810 (12)	0.0556 (5)	
H4	0.7555	0.4850	0.2603	0.083*	
O5	0.73100 (7)	0.86793 (11)	0.28224 (11)	0.0593 (5)	
O6	0.71141 (6)	0.94807 (11)	0.38534 (10)	0.0459 (4)	
O7	0.64163 (8)	0.93250 (12)	0.45856 (13)	0.0661 (5)	
H7	0.6652	0.9388	0.4344	0.099*	
O8	0.60196 (8)	0.81822 (13)	0.50512 (13)	0.0807 (6)	
C1	1.07715 (9)	0.16650 (16)	0.69809 (14)	0.0398 (6)	
H1	1.1077	0.1718	0.7597	0.048*	
C2	1.08895 (10)	0.11114 (17)	0.63505 (17)	0.0456 (6)	
H2	1.1267	0.0809	0.6543	0.055*	
C3	1.04430 (10)	0.10193 (16)	0.54458 (17)	0.0424 (6)	
H3	1.0517	0.0658	0.5016	0.051*	
C4	0.98738 (9)	0.14703 (15)	0.51668 (15)	0.0344 (6)	
C5	0.93748 (11)	0.13995 (16)	0.42380 (15)	0.0437 (6)	
H9	0.9429	0.1063	0.3775	0.052*	
C6	0.88307 (10)	0.18152 (17)	0.40309 (14)	0.0456 (6)	
H8	0.8508	0.1735	0.3433	0.055*	
C7	0.87341 (10)	0.23763 (16)	0.47020 (14)	0.0347 (6)	
C8	0.81794 (10)	0.28303 (17)	0.45171 (15)	0.0481 (7)	
H10	0.7846	0.2781	0.3925	0.058*	
C9	0.81285 (10)	0.33462 (17)	0.52063 (17)	0.0483 (7)	
H11	0.7759	0.3641	0.5093	0.058*	
C10	0.86370 (10)	0.34262 (16)	0.60827 (15)	0.0416 (6)	
H12	0.8598	0.3785	0.6545	0.050*	
C11	0.92208 (9)	0.24801 (15)	0.56050 (14)	0.0284 (5)	
C12	0.97938 (8)	0.20154 (15)	0.58436 (13)	0.0287 (5)	
C13	0.94314 (9)	0.42376 (17)	0.86098 (14)	0.0423 (6)	
H13	0.9339	0.3659	0.8795	0.051*	
C14	0.92679 (10)	0.50594 (19)	0.89267 (17)	0.0535 (7)	
H14	0.9068	0.5024	0.9311	0.064*	
C15	0.94016 (10)	0.59170 (18)	0.86711 (16)	0.0543 (7)	
H15	0.9296	0.6470	0.8882	0.065*	
C16	0.97025 (9)	0.59569 (17)	0.80814 (15)	0.0383 (6)	
C17	0.98481 (8)	0.50971 (16)	0.77988 (13)	0.0288 (5)	
C18	0.98564 (9)	0.68215 (17)	0.77834 (14)	0.0504 (7)	
H18	0.9760	0.7392	0.7978	0.061*	
C19	0.66694 (8)	0.59686 (16)	0.41710 (14)	0.0303 (5)	
C20	0.64826 (8)	0.67726 (16)	0.44598 (14)	0.0365 (6)	
H20	0.6259	0.6701	0.4805	0.044*	
C21	0.66067 (9)	0.76847 (16)	0.42678 (14)	0.0302 (5)	
C22	0.69433 (8)	0.77913 (15)	0.37392 (13)	0.0273 (5)	
C23	0.71376 (8)	0.69725 (17)	0.34589 (12)	0.0280 (5)	
H23	0.7367	0.7037	0.3122	0.034*	
C24	0.70075 (8)	0.60660 (16)	0.36553 (13)	0.0270 (5)	
C25	0.71945 (9)	0.51885 (18)	0.33192 (14)	0.0325 (6)	
C26	0.64675 (11)	0.50354 (17)	0.44011 (17)	0.0394 (6)	
C27	0.63255 (10)	0.84283 (18)	0.46660 (17)	0.0454 (6)	
C28	0.71315 (9)	0.87133 (16)	0.34430 (16)	0.0351 (6)	

### Atomic displacement parameters (Å^2^)

**Table:**

	*U*^11^	*U*^22^	*U*^33^	*U*^12^	*U*^13^	*U*^23^
Ni1	0.0374 (2)	0.0251 (3)	0.0303 (2)	0.000	0.01641 (19)	0.000
N1	0.0359 (10)	0.0267 (13)	0.0322 (10)	0.0028 (9)	0.0161 (9)	0.0017 (9)
N2	0.0349 (10)	0.0291 (12)	0.0354 (10)	0.0031 (9)	0.0171 (9)	0.0028 (10)
N3	0.0416 (10)	0.0303 (13)	0.0327 (10)	−0.0019 (9)	0.0248 (9)	0.0017 (10)
O1	0.0665 (11)	0.0350 (12)	0.0542 (10)	−0.0138 (9)	0.0289 (10)	0.0063 (10)
O1W	0.112 (3)	0.062 (3)	0.127 (3)	0.000	−0.001 (3)	0.000
O2	0.0499 (10)	0.0400 (12)	0.0752 (12)	−0.0150 (9)	0.0251 (10)	0.0019 (11)
O3	0.0644 (10)	0.0244 (10)	0.0554 (10)	0.0035 (9)	0.0343 (9)	0.0033 (9)
O4	0.0908 (12)	0.0336 (11)	0.0732 (12)	0.0046 (10)	0.0643 (10)	−0.0030 (10)
O5	0.1064 (13)	0.0326 (11)	0.0727 (11)	−0.0077 (10)	0.0704 (11)	0.0013 (10)
O6	0.0653 (10)	0.0225 (9)	0.0604 (10)	−0.0061 (8)	0.0373 (9)	−0.0044 (9)
O7	0.0981 (14)	0.0290 (12)	0.1078 (15)	0.0010 (11)	0.0788 (12)	−0.0063 (12)
O8	0.1316 (15)	0.0434 (13)	0.1301 (16)	−0.0002 (12)	0.1151 (14)	−0.0050 (13)
C1	0.0394 (13)	0.0362 (17)	0.0406 (13)	0.0039 (12)	0.0147 (12)	−0.0007 (13)
C2	0.0487 (15)	0.0356 (17)	0.0616 (16)	0.0055 (13)	0.0327 (14)	−0.0006 (15)
C3	0.0641 (16)	0.0268 (15)	0.0555 (16)	−0.0045 (13)	0.0438 (15)	−0.0063 (14)
C4	0.0463 (14)	0.0269 (15)	0.0367 (13)	−0.0076 (11)	0.0246 (13)	−0.0005 (12)
C5	0.0723 (17)	0.0308 (16)	0.0377 (14)	−0.0149 (14)	0.0331 (15)	−0.0078 (13)
C6	0.0570 (15)	0.0426 (18)	0.0281 (12)	−0.0147 (14)	0.0106 (12)	−0.0014 (14)
C7	0.0457 (14)	0.0275 (15)	0.0303 (13)	−0.0064 (12)	0.0163 (12)	0.0056 (12)
C8	0.0427 (15)	0.046 (2)	0.0409 (14)	−0.0040 (13)	0.0051 (12)	0.0069 (14)
C9	0.0388 (14)	0.0433 (19)	0.0552 (15)	0.0050 (12)	0.0141 (14)	0.0066 (15)
C10	0.0451 (14)	0.0346 (17)	0.0469 (15)	0.0052 (12)	0.0220 (13)	0.0028 (13)
C11	0.0390 (13)	0.0206 (14)	0.0283 (12)	−0.0020 (10)	0.0175 (11)	0.0025 (11)
C12	0.0374 (12)	0.0224 (14)	0.0297 (12)	−0.0040 (11)	0.0180 (11)	0.0014 (11)
C13	0.0556 (14)	0.0351 (17)	0.0463 (14)	−0.0044 (13)	0.0318 (13)	0.0006 (14)
C14	0.0743 (17)	0.046 (2)	0.0668 (17)	0.0042 (15)	0.0549 (15)	−0.0026 (17)
C15	0.0809 (17)	0.0329 (17)	0.0708 (17)	0.0069 (15)	0.0533 (16)	−0.0060 (16)
C16	0.0501 (14)	0.0302 (16)	0.0444 (14)	0.0013 (12)	0.0298 (13)	−0.0029 (13)
C17	0.0332 (13)	0.0258 (14)	0.0302 (13)	−0.0002 (10)	0.0165 (11)	−0.0007 (12)
C18	0.0761 (17)	0.0225 (14)	0.0686 (18)	0.0042 (13)	0.0465 (14)	−0.0031 (15)
C19	0.0350 (12)	0.0241 (14)	0.0345 (13)	−0.0021 (11)	0.0177 (11)	−0.0004 (12)
C20	0.0473 (13)	0.0304 (16)	0.0461 (13)	−0.0033 (12)	0.0338 (12)	−0.0007 (14)
C21	0.0369 (12)	0.0248 (15)	0.0339 (12)	0.0003 (11)	0.0201 (11)	−0.0010 (12)
C22	0.0313 (12)	0.0238 (15)	0.0258 (12)	−0.0019 (10)	0.0117 (10)	0.0007 (11)
C23	0.0308 (11)	0.0329 (15)	0.0235 (11)	−0.0006 (11)	0.0149 (9)	−0.0003 (12)
C24	0.0332 (12)	0.0230 (14)	0.0256 (11)	0.0025 (10)	0.0136 (10)	−0.0012 (11)
C25	0.0355 (13)	0.0333 (16)	0.0285 (13)	0.0013 (12)	0.0140 (11)	−0.0007 (13)
C26	0.0517 (16)	0.0263 (16)	0.0506 (16)	0.0014 (13)	0.0320 (15)	−0.0006 (15)
C27	0.0636 (16)	0.0265 (16)	0.0581 (16)	0.0019 (13)	0.0380 (14)	−0.0019 (15)
C28	0.0419 (13)	0.0239 (14)	0.0392 (14)	0.0008 (11)	0.0176 (12)	0.0037 (11)

### Geometric parameters (Å, °)

**Table:**

Ni1—N2^i^	2.0740 (15)	C6—C7	1.426 (3)
Ni1—N2	2.0740 (15)	C6—H8	0.9300
Ni1—N1	2.0809 (16)	C7—C8	1.398 (3)
Ni1—N1^i^	2.0809 (16)	C7—C11	1.401 (2)
Ni1—N3^i^	2.0943 (17)	C8—C9	1.363 (3)
Ni1—N3	2.0943 (17)	C8—H10	0.9300
N1—C1	1.335 (2)	C9—C10	1.393 (3)
N1—C12	1.362 (2)	C9—H11	0.9300
N2—C10	1.326 (2)	C10—H12	0.9300
N2—C11	1.365 (2)	C11—C12	1.426 (2)
N3—C13	1.330 (2)	C13—C14	1.392 (3)
N3—C17	1.361 (2)	C13—H13	0.9300
O1—C26	1.324 (2)	C14—C15	1.363 (3)
O1—H5	0.8200	C14—H14	0.9300
O1W—H1WA	0.80 (2)	C15—C16	1.417 (3)
O2—C26	1.210 (2)	C15—H15	0.9300
O3—C25	1.200 (2)	C16—C17	1.395 (3)
O4—C25	1.324 (2)	C16—C18	1.419 (3)
O4—H4	0.8200	C17—C17^i^	1.433 (3)
O5—C28	1.237 (2)	C18—C18^i^	1.357 (3)
O6—C28	1.276 (2)	C18—H18	0.9300
O7—C27	1.304 (3)	C19—C20	1.375 (3)
O7—H7	0.8200	C19—C24	1.395 (2)
O8—C27	1.200 (2)	C19—C26	1.508 (3)
C1—C2	1.394 (3)	C20—C21	1.389 (3)
C1—H1	0.9300	C20—H20	0.9300
C2—C3	1.366 (3)	C21—C22	1.411 (2)
C2—H2	0.9300	C21—C27	1.532 (3)
C3—C4	1.400 (3)	C22—C23	1.395 (3)
C3—H3	0.9300	C22—C28	1.523 (3)
C4—C12	1.402 (2)	C23—C24	1.389 (3)
C4—C5	1.435 (3)	C23—H23	0.9300
C5—C6	1.344 (3)	C24—C25	1.498 (3)
C5—H9	0.9300		
			
N2^i^—Ni1—N2	172.98 (11)	C9—C10—H12	118.5
N2^i^—Ni1—N1	95.24 (6)	N2—C11—C7	122.64 (18)
N2—Ni1—N1	80.02 (7)	N2—C11—C12	117.26 (18)
N2^i^—Ni1—N1^i^	80.02 (7)	C7—C11—C12	120.10 (19)
N2—Ni1—N1^i^	95.24 (6)	N1—C12—C4	123.05 (18)
N1—Ni1—N1^i^	96.17 (9)	N1—C12—C11	117.05 (18)
N2^i^—Ni1—N3^i^	94.15 (6)	C4—C12—C11	119.90 (18)
N2—Ni1—N3^i^	91.25 (6)	N3—C13—C14	123.0 (2)
N1—Ni1—N3^i^	92.43 (6)	N3—C13—H13	118.5
N1^i^—Ni1—N3^i^	170.00 (7)	C14—C13—H13	118.5
N2^i^—Ni1—N3	91.25 (6)	C15—C14—C13	119.6 (2)
N2—Ni1—N3	94.15 (6)	C15—C14—H14	120.2
N1—Ni1—N3	170.00 (7)	C13—C14—H14	120.2
N1^i^—Ni1—N3	92.44 (6)	C14—C15—C16	119.3 (2)
N3^i^—Ni1—N3	79.49 (9)	C14—C15—H15	120.3
C1—N1—C12	117.49 (17)	C16—C15—H15	120.3
C1—N1—Ni1	129.83 (14)	C17—C16—C15	117.0 (2)
C12—N1—Ni1	112.40 (13)	C17—C16—C18	120.33 (19)
C10—N2—C11	117.75 (17)	C15—C16—C18	122.7 (2)
C10—N2—Ni1	129.68 (15)	N3—C17—C16	123.54 (18)
C11—N2—Ni1	112.50 (12)	N3—C17—C17^i^	117.22 (11)
C13—N3—C17	117.48 (19)	C16—C17—C17^i^	119.24 (13)
C13—N3—Ni1	129.48 (16)	C18^i^—C18—C16	120.44 (12)
C17—N3—Ni1	113.03 (12)	C18^i^—C18—H18	119.8
C26—O1—H5	109.5	C16—C18—H18	119.8
C25—O4—H4	109.5	C20—C19—C24	118.5 (2)
C27—O7—H7	109.5	C20—C19—C26	117.07 (18)
N1—C1—C2	123.10 (19)	C24—C19—C26	124.4 (2)
N1—C1—H1	118.5	C19—C20—C21	124.15 (19)
C2—C1—H1	118.5	C19—C20—H20	117.9
C3—C2—C1	119.1 (2)	C21—C20—H20	117.9
C3—C2—H2	120.4	C20—C21—C22	117.8 (2)
C1—C2—H2	120.4	C20—C21—C27	111.71 (17)
C2—C3—C4	119.9 (2)	C22—C21—C27	130.5 (2)
C2—C3—H3	120.1	C23—C22—C21	117.7 (2)
C4—C3—H3	120.1	C23—C22—C28	115.15 (18)
C3—C4—C12	117.33 (19)	C21—C22—C28	127.2 (2)
C3—C4—C5	123.7 (2)	C24—C23—C22	123.67 (17)
C12—C4—C5	118.92 (19)	C24—C23—H23	118.2
C6—C5—C4	120.6 (2)	C22—C23—H23	118.2
C6—C5—H9	119.7	C23—C24—C19	118.2 (2)
C4—C5—H9	119.7	C23—C24—C25	123.52 (19)
C5—C6—C7	121.9 (2)	C19—C24—C25	118.3 (2)
C5—C6—H8	119.1	O3—C25—O4	124.2 (2)
C7—C6—H8	119.1	O3—C25—C24	121.9 (2)
C8—C7—C11	117.5 (2)	O4—C25—C24	113.8 (2)
C8—C7—C6	124.0 (2)	O2—C26—O1	120.6 (2)
C11—C7—C6	118.55 (19)	O2—C26—C19	121.8 (2)
C9—C8—C7	119.8 (2)	O1—C26—C19	117.2 (2)
C9—C8—H10	120.1	O8—C27—O7	120.0 (2)
C7—C8—H10	120.1	O8—C27—C21	119.7 (2)
C8—C9—C10	119.2 (2)	O7—C27—C21	120.2 (2)
C8—C9—H11	120.4	O5—C28—O6	122.7 (2)
C10—C9—H11	120.4	O5—C28—C22	117.9 (2)
N2—C10—C9	123.1 (2)	O6—C28—C22	119.40 (19)
N2—C10—H12	118.5		
			
N2^i^—Ni1—N1—C1	−3.79 (18)	Ni1—N1—C12—C11	−7.6 (2)
N2—Ni1—N1—C1	−178.55 (18)	C3—C4—C12—N1	0.2 (3)
N1^i^—Ni1—N1—C1	−84.29 (18)	C5—C4—C12—N1	179.90 (19)
N3^i^—Ni1—N1—C1	90.60 (18)	C3—C4—C12—C11	−179.05 (18)
N3—Ni1—N1—C1	126.5 (4)	C5—C4—C12—C11	0.6 (3)
N2^i^—Ni1—N1—C12	−177.45 (13)	N2—C11—C12—N1	1.8 (3)
N2—Ni1—N1—C12	7.79 (13)	C7—C11—C12—N1	−177.96 (19)
N1^i^—Ni1—N1—C12	102.05 (14)	N2—C11—C12—C4	−178.87 (17)
N3^i^—Ni1—N1—C12	−83.05 (13)	C7—C11—C12—C4	1.3 (3)
N3—Ni1—N1—C12	−47.1 (4)	C17—N3—C13—C14	0.6 (3)
N2^i^—Ni1—N2—C10	128.53 (18)	Ni1—N3—C13—C14	179.91 (17)
N1—Ni1—N2—C10	176.55 (19)	N3—C13—C14—C15	−0.5 (4)
N1^i^—Ni1—N2—C10	81.19 (18)	C13—C14—C15—C16	0.4 (4)
N3^i^—Ni1—N2—C10	−91.19 (18)	C14—C15—C16—C17	−0.3 (3)
N3—Ni1—N2—C10	−11.64 (19)	C14—C15—C16—C18	179.9 (2)
N2^i^—Ni1—N2—C11	−54.87 (13)	C13—N3—C17—C16	−0.5 (3)
N1—Ni1—N2—C11	−6.84 (13)	Ni1—N3—C17—C16	180.00 (16)
N1^i^—Ni1—N2—C11	−102.20 (13)	C13—N3—C17—C17^i^	179.96 (18)
N3^i^—Ni1—N2—C11	85.41 (14)	Ni1—N3—C17—C17^i^	0.5 (3)
N3—Ni1—N2—C11	164.96 (13)	C15—C16—C17—N3	0.4 (3)
N2^i^—Ni1—N3—C13	−85.57 (16)	C18—C16—C17—N3	−179.79 (19)
N2—Ni1—N3—C13	89.94 (16)	C15—C16—C17—C17^i^	179.9 (2)
N1—Ni1—N3—C13	143.9 (3)	C18—C16—C17—C17^i^	−0.3 (4)
N1^i^—Ni1—N3—C13	−5.50 (16)	C17—C16—C18—C18^i^	0.4 (4)
N3^i^—Ni1—N3—C13	−179.55 (19)	C15—C16—C18—C18^i^	−179.9 (2)
N2^i^—Ni1—N3—C17	93.81 (14)	C24—C19—C20—C21	0.3 (3)
N2—Ni1—N3—C17	−90.68 (14)	C26—C19—C20—C21	−177.1 (2)
N1—Ni1—N3—C17	−36.8 (4)	C19—C20—C21—C22	0.3 (3)
N1^i^—Ni1—N3—C17	173.87 (14)	C19—C20—C21—C27	178.78 (19)
N3^i^—Ni1—N3—C17	−0.18 (10)	C20—C21—C22—C23	−1.1 (3)
C12—N1—C1—C2	1.6 (3)	C27—C21—C22—C23	−179.2 (2)
Ni1—N1—C1—C2	−171.78 (16)	C20—C21—C22—C28	179.9 (2)
N1—C1—C2—C3	−0.6 (3)	C27—C21—C22—C28	1.7 (3)
C1—C2—C3—C4	−0.7 (3)	C21—C22—C23—C24	1.3 (3)
C2—C3—C4—C12	0.8 (3)	C28—C22—C23—C24	−179.49 (18)
C2—C3—C4—C5	−178.8 (2)	C22—C23—C24—C19	−0.8 (3)
C3—C4—C5—C6	176.8 (2)	C22—C23—C24—C25	177.38 (18)
C12—C4—C5—C6	−2.8 (3)	C20—C19—C24—C23	−0.1 (3)
C4—C5—C6—C7	3.0 (3)	C26—C19—C24—C23	177.1 (2)
C5—C6—C7—C8	179.3 (2)	C20—C19—C24—C25	−178.30 (19)
C5—C6—C7—C11	−1.0 (3)	C26—C19—C24—C25	−1.2 (3)
C11—C7—C8—C9	−0.2 (3)	C23—C24—C25—O3	179.5 (2)
C6—C7—C8—C9	179.5 (2)	C19—C24—C25—O3	−2.4 (3)
C7—C8—C9—C10	1.2 (3)	C23—C24—C25—O4	−2.7 (3)
C11—N2—C10—C9	−0.8 (3)	C19—C24—C25—O4	175.48 (17)
Ni1—N2—C10—C9	175.62 (17)	C20—C19—C26—O2	83.8 (3)
C8—C9—C10—N2	−0.6 (3)	C24—C19—C26—O2	−93.4 (3)
C10—N2—C11—C7	1.8 (3)	C20—C19—C26—O1	−89.2 (2)
Ni1—N2—C11—C7	−175.25 (16)	C24—C19—C26—O1	93.6 (2)
C10—N2—C11—C12	−177.99 (18)	C20—C21—C27—O8	−2.9 (3)
Ni1—N2—C11—C12	5.0 (2)	C22—C21—C27—O8	175.3 (2)
C8—C7—C11—N2	−1.3 (3)	C20—C21—C27—O7	177.2 (2)
C6—C7—C11—N2	179.00 (18)	C22—C21—C27—O7	−4.5 (4)
C8—C7—C11—C12	178.51 (18)	C23—C22—C28—O5	15.8 (3)
C6—C7—C11—C12	−1.2 (3)	C21—C22—C28—O5	−165.12 (18)
C1—N1—C12—C4	−1.4 (3)	C23—C22—C28—O6	−162.56 (18)
Ni1—N1—C12—C4	173.10 (15)	C21—C22—C28—O6	16.5 (3)
C1—N1—C12—C11	177.87 (17)		

Symmetry codes: (i) −*x*+2, *y*, −*z*+3/2.

### Hydrogen-bond geometry (Å, °)

**Table:**

*D*—H···*A*	*D*—H	H···*A*	*D*···*A*	*D*—H···*A*
O4—H4···O5^ii^	0.82	1.87	2.690 (2)	177.
O1—H5···O6^iii^	0.82	1.81	2.624 (2)	173.
O7—H7···O6	0.82	1.63	2.4450 (19)	178.
O1W—H1WA···O2^iv^	0.80 (2)	2.10 (3)	2.866 (3)	158 (4)

Symmetry codes: (ii) −*x*+3/2, *y*−1/2, −*z*+1/2; (iii) −*x*+3/2, −*y*+3/2, −*z*+1; (iv) −*x*+1, *y*, −*z*+1/2.
